# Experiences of coping and adjusting to lower limb prosthesis use in the Eastern Cape province

**DOI:** 10.4102/ajod.v14i0.1706

**Published:** 2025-10-15

**Authors:** Noluvuyo Seti, Lieketseng Y. Ned

**Affiliations:** 1 Division of Disability and Rehabilitation Studies, Faculty of Medicine and Health Sciences, Stellenbosch University, Cape Town, South Africa

**Keywords:** prosthesis, lower limb amputation, rehabilitation, OR Tambo District, South Africa

## Abstract

**Background:**

Lower limb amputation is the surgical removal of a limb, typically because of trauma or chronic illness. A prosthesis can aid recovery, but in South Africa’s Eastern Cape, access to prosthetic rehabilitation services remains limited.

**Objectives:**

The aim of this study was to describe and explore experiences of lower limb prosthetic users in coping and adjusting to prosthesis use in the OR Tambo District.

**Method:**

Using interpretative phenomenological analysis, this qualitative study purposefully sampled five lower limb prosthesis users. Data were collected through semi-structured face-to-face interviews conducted in participants’ homes. The interviews were transcribed and analysed using interpretive phenomenological analysis, allowing for an in-depth exploration of themes related to adaptation and coping.

**Results:**

Three themes emerged: (1) facing psychological and identity adjustments, participants reported a range of emotions from shock to happiness, (2) navigating daily societal realities and perceptions, emphasising the influence of stigma and support on prosthesis user experience, and (3) learning to cope and receive support, focusing on adaptive coping strategies and support systems, including family and the community.

**Conclusion:**

The findings underscore the need for comprehensive and accessible rehabilitation services that address physical, emotional and social challenges. Tailored prosthesis designs for rural environments and community education programmes to reduce stigma are essential for improving user outcomes.

**Contribution:**

This study advocates for holistic prosthetic care, emphasising ongoing support and proactive engagement with users’ experiences to improve quality of life and promote independence.

## Introduction

Lower limb amputation (LLA) is the surgical removal of a limb because of trauma or chronic disease. Holzer et al. (2014) highlighted that individuals who have undergone LLA must adjust mentally, physically and socially to significant changes in their appearance and functioning. People’s responses to limb loss and prosthesis use are both complex and unique, shaped by a range of personal, clinical, social, physical and environmental factors (Aydin et al. 2021). Individuals frequently encounter psychological and social difficulties, including depression, hopelessness, low self-esteem, fatigue, anxiety, frustration, guilt, fear about their family’s future and, in some cases, even suicidal thoughts because of difficulties adapting to their new circumstances (Dadkhah et al. 2013). Beyond the immediate physical and emotional challenges, LLA often leads to long-term socio-economic consequences (Lee et al. 2020) as individuals may face job loss, financial instability and social isolation because of their reduced mobility and dependence on others. A study conducted in Portugal by Pereira et al. (2018) revealed that post-amputation life satisfaction was generally low. However, the most effective coping mechanism reported was the proactive adjustment to the new reality, whereas substance use was the least effective strategy.

Following the amputation, people frequently require a prosthetic device to assist them to regain mobility and improve their quality of life. A lower limb prosthesis is a custom-made artificial device designed to replace an amputated lower limb. In individuals with LLA, the purpose of prosthesis intervention aims to regain ambulation as well as general functioning (Haywood 2020). The artificial limb supports people to regain their mobility after having their lower limbs amputated and therefore can lead to increased independence (Morgan et al. 2022; Seth et al. 2022). Unfortunately, in the Eastern Cape province and broadly in South Africa, prosthetics and rehabilitation facilities remain inadequate, and numerous individuals are unable to progress to the stage of receiving the prosthesis for recovery (Ennion & Johannesson 2018). Currently, the province has only three prosthetic centres (in Umtata, East London and Port Elizabeth, respectively). Drawing from her experience of working at Bedford Orthopaedic Hospital in Umtata, the first author learnt that, as prosthetists, they only issue prosthetic devices and do not always carry out follow-ups. As Ennion and Manig (2019) argue, the distribution of rehabilitative and artificial limbs within the South African public health sector is hampered by employee shortages, a scarcity of referrals, education and follow-up with patients, a shortage of qualified rehabilitation experts, insufficient training for healthcare professionals working in rural areas and a lack of funds.

Individuals who have undergone an LLA possess numerous expectations regarding how a prosthetic device is going to impact their daily lives. Research by Mattick et al. (2022) and Ostler, Ellis-Hill and Donovan-Hall (2014) reports that participants expected that the prosthesis would allow them to regain their sense of ‘normalcy’. Their return to ‘normalcy’ was characterised by a physical appearance that corresponded with their sense of self, capacity and perception, among others, prior to the amputation. This included having the capacity to perform daily duties such as cleaning and preparing meals, along with the ability to work and provide for family members. Cultural beliefs and perceptions were found to influence how individuals experience prosthesis use (Mattick et al. 2022). Studies documenting the experiences of users do not vary much from these expectations. For example, a study conducted in Mpumalanga reports that prosthetic assistance proved to be important in enabling participants to engage in everyday duties while continuing to be active citizens in their communities (Ennion & Manig 2019). Another study in Kenya revealed that participants saw the possibility of receiving an artificial limb as an optimistic representation of a more promising future (Mattick et al. 2022). The prosthesis allowed these individuals to resume previous activities, integrate into society and work and be less dependent on others. Similarly, Murray and Forshaw’s (2013) study in the United Kingdom showed that starting to utilise an artificial limb was a vital aspect of restoring a desired sense of self, including returning to employment or participating in everyday activities, like driving a car. The participants’ self-worth was subsequently derived through the self-efficacy and freedom that their prosthesis use enabled or facilitated.

Individuals that use a prosthetic must cope with and adjust to a variety of aspects, including shoe alternatives, reactions from relatives and close companions, sexuality, phantom sensations, stump sensibility, emotions, running, walking backwards, footsteps and distance covered by walking, comfort with the prosthesis, weight and dependability (Norlyk et al. 2016). Aydin et al.’s (2021) research indicates that active and task-oriented coping strategies, such as problem-solving, promote positive psychosocial adjustments, while emotion-focused coping and cognitive disengagement are associated with anxiety, depression and externalised hostility, impacting negatively on acceptance of disability. A study conducted in Portugal reported that individuals’ acceptance and proactive adjustments remained the most utilised coping mechanisms, while substance use, such as alcohol or drug consumption to manage emotional distress or physical discomfort, was the least commonly utilised (Pereira et al. 2018).

Considering methods of coping as a significant measurement, particularly in a rehabilitation programme, may improve patients’ quality of life and overall well-being (Šosterič, Burger & Vidmar 2020). Participants in Ostler et al.’s (2014) study in the United Kingdom addressed the necessity of having a positive mindset during rehabilitation, implying that if this positive mindset failed, there would be negative effects. This qualitative metasynthesis by Ostler et al. (2014) highlighted the societal variable as playing a vital role in coping and adjusting. For example, societal acceptance, such as witnessing a positive attitude from friends and relatives, was cited as a significant contributor to coping and adjusting.

The experiences of prosthesis users in coping and adjusting to prosthetic use are both complex and multifaceted, influenced by various physical, psychological and social factors. Yet, it remains under-researched. Specifically, there remains inadequate research about lower limb prosthesis users’ experiences of coping and adjusting throughout the African continent, including the Eastern Cape. Documenting these experiences will bring to the forefront much-needed information from prosthetic users’ perspectives, which may positively influence service provision and follow-up support. Therefore, this study sought to answer the question: *What are lower limb prosthetic users’ experiences in coping and adjusting to prosthesis use in the OR Tambo District, Eastern Cape?*

## Research methods and design

This study followed a qualitative methodology. The chosen study design was interpretive phenomenological analysis (IPA) – a design that seeks to provide perspectives on how a given individual, in a specific setting, makes meaning of a certain circumstance, rather than creating a generalisation of findings. Interpretive phenomenological analysis is rooted in hermeneutic phenomenology, which focuses not only on describing lived experiences (Smith & Osborn 2007). As Alase (2017) posits, IPA is often suitable for qualitative research when the aim is to explore and understand individuals’ lived experiences, perceptions and meanings attributed to a specific phenomenon. It is particularly valuable in fields such as health sciences, where researchers seek rich insights into subjective experiences of participants. This design was therefore chosen to uncover the experiences of individuals who are adapting to and using prosthetic limbs, with a focus on understanding their emotional, social and physical challenges and coping strategies throughout the process.

### Research setting

The research was conducted in the OR Tambo District of the Eastern Cape; it is one of the seven districts of the Eastern Cape province of South Africa. This district is characterised by its rural and urban mix, with many communities living in locations that are remote and challenging for access to healthcare facilities and support services.

OR Tambo District, within the Wild Coast region, is one of the four integrated Sustained Rural Development Programme sites in the Eastern Cape province and is one of the seven districts in the province. In 2019, the population of the district was 1,514,306 persons in 2020 (OR Tambo District Municipality 2020). Bedford Orthopaedic Hospital is the only public hospital offering prosthetic services in the OR Tambo District, and it serves clients from the surrounding former Transkei districts. As Manig (2018) noted, 128 amputations of the lower limbs were conducted in the OR Tambo District from 2015 to 2017. Anecdotal evidence also shows that, on an annual basis, around 120 prostheses are issued at Bedford Orthopaedic Hospital (Personal communication, staff working in the Medical Orthotics and Prosthetics Department). It is, however, not clear how much of these are primarily based in the OR Tambo District.

The participants of this study come from diverse residential areas such as the Ncambele, Ngxwala and Mpumazi locations, reflecting the range from rural, sparsely populated areas to urbanised settings. The varied geography and socio-economic conditions of the OR Tambo District provided a comprehensive backdrop for understanding the experiences and challenges faced by individuals using prosthetics. This setting offers a unique perspective on the accessibility and adaptation of prosthesis usage in rural versus urban environments, highlighting the disparities in available resources, support systems and the physical environment for individuals in different parts of the district.

### Study population and size

The study population comprised adults in the OR Tambo District who had undergone LLA and had been using a prosthesis for at least 1 year. Participants were included if they met the following inclusion criteria: (1) adults (18+ years) who had been using a lower limb prosthesis for at least a year, and (2) were long-term residents of the OR Tambo District to ensure consistency in prosthesis components and services. Individuals who had received their prosthesis services from the researcher or those who recently moved to the OR Tambo District or had obtained their prosthesis from the private sector were excluded to maintain study consistency.

Initially, we planned for 12 participants. Pietkiewicz and Smith (2014) posit that IPA studies have been published with sample sizes ranging from 1 participant to 15 participants. While larger samples are possible, they are less common.

Recruiting participants for this study was challenging, as the first author had to rely on phone calls. Many people in rural areas experience poor network coverage, and some change their numbers – this made the process difficult, as many calls often went to voicemail. The first author persistently tried to reach them telephonically, and even when they answered, the conversation was frequently unclear because of poor connectivity issues. From Bedford Orthopaedic Hospital, we obtained a list of 18 individuals with their contact details. Unfortunately, two of them had passed away, and four were not interested in participating. Two cell phone numbers were incorrect, and five calls consistently went to voicemail, leaving us with only five participants. These participants were diverse in terms of age, gender and occupation, but all shared the experience of coping and adapting to life with a prosthesis. These five participants met the inclusion criteria and consented during the data collection period. The final sample size, although smaller than initially anticipated, was sufficient for a detailed qualitative analysis. Data saturation was also reached after five interviews, as no new themes were emerging.

First contact with potential participants was made by the first author, with assistance from the Head of department at Bedford Orthopaedic Hospital. The first author also approached participants during their follow-up visits in the hospital. The study was explained in detail, including its purpose, procedures, risks and benefits. Those who showed interest were given time to ask questions, and written consent was obtained prior to participation.

### Data collection methods and analysis

Semi-structured interviews were the most suitable interview type for an IPA study (Alase 2017). The interviews were conducted face to face at the participants’ homes, where they felt more comfortable and at ease. This approach made the interviews more convenient for participants, as they did not need to travel to other locations. Interviews lasted between 45 min and 60 min at the participants’ homes and were conducted in isiXhosa and English, based on participants’ preferences. The length of an IPA interview varies widely, but in this study, each interview lasted from 45 min to 60 min. As Smith and Osborn (2007) and Pietkiewicz and Smith (2014) put it, most IPA interviews last an hour or more; however, shorter or longer interviews occur based on the depth of exploration needed. Each participant received an R200.00 Shoprite (supermarket) voucher as a token of appreciation for their time and participation.

A self-developed interview guide was the data collection tool for this study. A self-developed interview guide can be customised to align precisely with specific research goals and questions, ensuring that the questions are directly relevant to the research aim. Previous literature (such as Ennion & Manig 2019) assisted and played a crucial role in developing the interview questions by providing a foundation of knowledge and insight into the topic of interest. Sample questions included: ‘Can you describe how your life has changed since using the prosthesis?’ and ‘What challenges did you face during adjustment?’ Further probing and prompting were done during the interview to get participants to delve deeper into the meaning of their experiences. The questions in this interview were exploratory (designed to gather more information, clarify responses or seek further details from the interviewee) and aimed to uncover the participant’s lived experiences of using a prosthesis.

### Analysis and data management

All interviews were audio-recorded and later transcribed. After transcribing, participants were asked to verify whether the transcripts accurately reflected their accounts.

The study followed the steps of the IPA approach, as outlined by Crist and Tanner (2003), Smith and Osborn (2007) and Pietkiewicz and Smith (2014), as follows:

- Initial reading and note taking – the analysis began with a thorough reading and rereading of each interview transcript while detailed notes were taken.
- Generation of initial codes – the coding process involved identifying and labelling meaningful segments of text (codes) related to the participants’ experiences and perspectives. These codes captured the essence of what the participants expressed.
- Grouping codes into themes – codes that shared common characteristics were organised and grouped into preliminary themes. This process involved looking for patterns and connections within the data.
- Refinement of themes – themes were revived and refined by cross-referencing them with the original transcripts and codes.
- Writing descriptive narratives – descriptive narratives were developed for each participant based on identified themes. This narrative provided a detailed account of the participant’s experiences.
- Cross-case analysis – themes and narratives were compared across participants to identify overarching patterns and differences in their experiences. The initial analysis, however, was conducted on a case-by-case basis.
- Writing up the results – a comprehensive report was developed, outlining the themes, narratives and findings from the analysis.

After this analysis, the first author presented findings to participants to ensure correct interpretation, as part of member checking. Regular discussions between the first author and the supervisor (second author) helped refine, validate and confirm the structure and interpretative findings. The second author also gave regular feedback on the different versions of analysed data.

Regarding data management, data were securely stored on Microsoft OneDrive (only the first author had the password, which helped to protect the account from unauthorised access) and a cloud platform (for backup). This safeguarded sensitive information, ensuring that only authorised personnel could access and manage the data, thereby enhancing data security and privacy.

### Ethical considerations

Ethical approval was first sought from the Stellenbosch University Health Research Ethics Committee (HREC) (Reference No: S23/10/254) and from the Eastern Cape Department of Health (EC_202401_019), and institutional permission was sought from Nelson Mandela Academic Hospital (EC_202402_008). Written informed consent was obtained from all participants, and confidentiality was maintained throughout.

## Results

Table 1 depicts the demographic details of participants who were included.

**TABLE 1:** Demographic details of participants.

Participants	Age	Gender	Residence type	Years using prosthesis
P1	27	Female	Rural	2.0
P2	49	Male	Rural	3.0
P3	33	Female	Urban	3.0
P4	45	Male	Rural	1.5
P5	65	Male	Rural	4.0

Following analysis, three themes describe the experiences of lower limb prosthesis users.

### Theme 1: Facing psychological and identity adjustments

The initial reaction of participants following the use of a prosthesis varied and revealed the profound emotional and psychological impact of adjusting to using an assistive device. Some participants actively sought ways to manage these emotions through professional counselling and family support. For example, Participant 1 (P1) mentioned that counselling was crucial in helping her come to terms with her new reality, as it provided a space to discuss her fears and frustration. P3 found motivation in helping others who were going through similar experiences to turn their emotional struggles into sources of strength and personal empowerment. For others, it was a moment of joy and newfound hope. In contrast, the experience was overwhelming and filled with sadness for other participants. These participants described feeling overwhelmed by the reality of relying on an artificial limb, which presented a drastic change in their sense of self and how they navigated the world. P1 mentioned that she struggled to get used to the prosthesis because she was ‘used to my own leg’, and now she ‘had to learn to rely on something foreign to me’. These support systems (including emotional support from family, encouragement from peers and community acceptance) were essential in helping participants adapt, offering both emotional support and practical help that alleviated their daily activities:

‘It was very difficult, sister. I was shocked, to be honest. Having to use an artificial leg instead of my own, it was something I never thought would happen to me.’ (P1, 27, female, rural)

‘The idea of relying on a prosthesis felt daunting, and I couldn’t shake the memories of what I had lost.’ (P3, 33, female, urban)

Despite the initial shock, some participants expressed feelings of relief and joy as they began to realise that the prosthesis could restore some degree of functional independence and mobility. As they gained more confidence in using the prosthesis, the emotional burden lessened, and they started to see the artificial limb as a tool that enabled them to engage in their daily lives with greater independence and ease. However, the adjustment process was not without its challenges, as many struggled with adapting to their new reality and the physical difficulties of learning to walk with a prosthesis. Over time, participants reported that they began to embody the prosthesis as part of their new identity:

‘I felt like my life was beginning to return to normal and I have accepted my new reality. My excitement came from knowing that I could regain my independence and start living like before.’ (P2, 49, male, rural)

‘Over time, though, I got used to it and learned to accept it as part of my life.’ (P3, 33, female, urban)

Participants expressed happiness at the newfound ability to walk unaided, after long periods of relying on crutches and wheelchairs. The emotional relief was deeply tied to the rediscovery of freedom and the ability to participate in activities that had previously seemed out of reach when they were using crutches and wheelchairs. For these participants, walking without assistance symbolised progress and a return to life as they once knew it. However, alongside these positive emotions, participants also experienced moments of doubt and frustration as they adjusted to the challenges of prosthesis use. Physical discomfort, such as pressure sores and the difficulties of navigating uneven surfaces, often reminded them of their limitations, briefly interrupting their progress. For instance, participant 4 shared: ‘After two years of use, I developed a painful sore at the end of the stump’. Despite these setbacks, the overarching emotional response remained one of optimism and determination, as participants focused on small victories and the long-term benefits of their prosthetic adaptation. This mix of joy and frustration highlights the emotional resilience required to navigate the transition from dependency to newfound independence:

‘I felt a sense of freedom again. I could walk without needing anyone’s help, and that gave me hope. It felt like I was normal again.’ (P1, 27, female, rural)

‘While I was mostly positive, there were moments of doubt, especially when I struggled to walk on uneven surface. I reminded myself of my goals and how far I’d come.’ (P2, 49, male, rural)

The use of a prosthesis brought significant changes in participants’ self-image and self-esteem, as they gradually regained their confidence. This renewed self-image allowed participants to engage more confidently with others and reclaim their roles within their families and communities. The independence gained from using the prosthesis was particularly empowering, as it reinforced their self-worth and provided a boost in self-esteem. Several participants initially struggled with body image issues, feeling incomplete or diminished after their amputation. Over time, they began to accept their bodies and appreciate their strength and capabilities. Being able to participate in activities without relying on others restored their dignity and respect, both in their own eyes and in the eyes of those around them. This transformation was gradual but profound, as participants noted a positive shift in how they viewed themselves and how others interacted with them:

‘Once I got used to the prosthesis and saw that I could still move and do things, I began to see my body in a new light.’ (P1, 27, female, rural)

‘Knowing that I can take care of myself has been empowering, and it restored my confidence in many ways.’ (P2, 49, male, rural)

### Theme 2: Navigating daily societal realities and perceptions

The experiences of participants were also shaped by external factors such as stigma and societal attitudes which they were subjected to. For those who faced stigma, avoiding public places initially seemed like the only solution. Those who encountered negative societal attitudes experienced being stared at or receiving negative comments, which had a profound impact on their self-image and introduced feelings of wanting to disengage from public life. These negative interactions often led to feelings of isolation and alienation, as some participants chose to limit their outings to avoid uncomfortable situations. With time, they learnt coping mechanisms, including focusing on their personal journey and seeking emotional support, which helped them reclaim their confidence and engage more freely in social spaces:

‘Whenever I went out, people would stare at me. Some would look at me with pity, while others would laugh and say I had a “doll’s leg”. It hurt a lot, and it made me feel like people didn’t see me the same way they used to. I dealt with it by staying home most of the time.’ (P1, 27, female, rural)

‘I do notice some pitying looks from strangers when I’m walking in the streets, but it doesn’t affect me much.’ (P3, 33, female, urban)

There are also participants who had not encountered any stigma or discrimination related to the prosthesis. In fact, they experienced positive societal attitudes, often receiving respect and admiration for their resilience and independence. The presence of supportive communities and the strength of their self-sufficiency played a crucial role in shaping these positive experiences. These participants noted that their positions of responsibility, such as serving as the community counsellor or actively participating in daily tasks, reinforced the respect they received from others. However, they were aware of the challenges faced by others in similar situations. This level of awareness made them more appreciative of the inclusive attitudes within their own communities:

‘Thankfully, I’ve never experienced any stigma or negative societal attitudes. In my community, people respect me and don’t discriminate against me because of the prosthesis.’ (P2, 49, male, rural)

‘Thankfully, I haven’t faced stigma. My community has been very supportive. I think my self-sufficiency has helped others view me positively.’ (P5, 65, male, rural)

The participants expressed how the prosthesis allowed them to regain control over household chores and personal activities, which in turn improved self-sufficiency and confidence. For some participants, the ability to carry out simple household tasks was a significant victory, marking a step towards reclaiming their independence. One participant described how being able to do daily chores, like cooking and cleaning, made them feel ‘whole again’ after a period of dependency on others. Overall, the prosthesis gave participants the freedom to engage more fully in daily life, manage their household and take pride in their independence. Although their activities sometimes required more thought and adaptations compared to before their amputation, the ability to perform tasks unaided reinforced their self-worth:

‘Mowing my lawn again was incredibly fulfilling. It symbolised my regained independence and ability to manage my home.’ (P2, 49, male, rural)

‘It made me feel capable and independent, showing me that I could still contribute to my home and not be reliant on others.’ (P5, 65, male, rural)

Participants also shared environmental and physical limitations, which played a significant role in shaping their experiences. For many, the transition from the controlled environments of hospitals and physiotherapy centres to their home environments posed unexpected difficulties. Uneven terrain, particularly in rural areas, made it harder for participants to navigate, affecting their emotional well-being and limiting their independence. In addition to environmental factors, physical complications such as pressure sores or other medical conditions hindered some participants’ ability to use the prosthesis effectively. While the prosthesis provided increased mobility, the transition to using it daily was fraught with physical and emotional challenges. Environmental factors, such as uneven ground in rural areas, significantly impacted participants’ ability to move freely and confidently, often leading to frustration and regression. However, participants employed various strategies, such as using walking sticks, to cope with these challenges and regain independence:

‘The biggest challenge for me was walking with the leg at home in the rural areas. At physiotherapy in Bedford, everything seemed easy because it was flat and well-prepared for people with prosthetics. But back home, the paths are rough and full of stairs.’ (P1, 27, female, rural)

‘Physically, walking at home was a struggle at first. The ground in my yard is uneven and stony, which made it difficult to move around with the prosthetic leg. Hospitals and physiotherapy settings are designed for easy movement, but that’s not the reality when you return home.’ (P2, 49, male, rural)

### Theme 3: Learning to cope and receive support

The availability of a support system played a crucial role in how participants adjusted to life with a prosthesis. While some received help from formal services like physiotherapy, most relied heavily on family and close friends for both emotional and practical support. This encouragement provided comfort during emotionally difficult periods and helped ease the physical burden of adaptation. Participants repeatedly credited their loved ones for motivating them and remaining present throughout their rehabilitation:

‘My wife was there for me every step of the way. She came to every appointment and kept me motivated when I wanted to give up.’ (P4, 45, male, rural)

‘My sister has been my rock throughout this journey. From hospital visits to just being there for emotional support, her presence has made a world of difference.’ (P5, 65, male, rural)

The personal growth and positive outcomes experienced by participants after using a prosthesis were marked by a sense of newfound independence. Participants expressed satisfaction at being able to regain purpose, contribute to their families and engage in meaningful activities that had previously seemed impossible. This emotional growth was deeply tied to the realisation that they could still lead fulfilling lives despite their physical limitation. For many participants, the use of a prosthesis was a turning point that enabled them to regain independence and reclaim their purpose in life. The opportunity to return to work, engage in sports or perform daily tasks without relying on others fostered personal growth and self-confidence. By overcoming the challenges of using a prosthesis, participants felt empowered and capable, often expressing pride in their achievement. Participant 2 found fulfilment returning to sports, particularly javelin, where setting a record meant both physical and emotional progress: ‘Setting a record felt like a true testament to my journey’. For another participant, a return to work meant a return to normal life:

‘Before that, I was just sitting at home feeling useless, but getting back into the workforce showed me that I could still have a normal life.’ (P1, 27, female, rural)

However, personal growth in this context does not always stem from personal achievements alone. For other participants, the process of using a prosthesis fostered a deeper connection to others, particularly through acts of support and encouragement. Participants found personal growth through helping others and becoming a source of motivation. By offering support and guidance, these individuals reinforced their own resilience while positively impacting others’ lives. Participants experienced positive growth by becoming sources of strength and encouragement for others, turning their personal challenges into opportunities for positive impact and emotional growth:

‘I’ve even been able to motivate other people who are going through similar experiences.’ (P3, 33, female, urban)

‘Taking on small jobs gave me a purpose that I thought I had lost. It felt good to contribute financially and prove to myself that I could still support my family despite my challenges.’ (P4, 45, male, rural)

The participants’ experiences highlight the complex and personal journey of adjusting to prosthesis use, where learning to cope requires patience, self-motivation and external support. Participants described the physical and emotional challenges they faced during the adaptation period. The experience of adjusting to a prosthesis, as described by participants, reflects a complex journey of learning to cope both physically and emotionally. Participants had to take the initiative in their adaptation process, often with minimal access to consistent rehabilitation services. Many spoke of the need for self-directed learning, emphasising the lack of accessible, consistent rehabilitation services. This meant teaching themselves basic skills like walking and balancing, while others benefitted from more formal support systems:

‘I adjusted to the prosthesis by practicing walking around the village. I wanted to become accustomed to it quickly, so I made sure to walk as much as possible.’ (P5, 65, male, rural)

‘I set small goals for myself, like walking a certain distance each day. Celebrating those little achievements helped keep me motivated.’ (P4, 45, male, rural)

These stories of gradual progress emphasise the importance of emotional and motivational support from others. For some, the support of physiotherapists, teachers or friends made a significant impact on their adjustment. Emotional support from friends, family and the broader community also emerged as critical for coping and adaptation. The role of social support extended beyond formal rehabilitation. For P3, returning to high school with a prosthesis was made easier by the community’s acceptance and encouragement:

‘My teachers were very encouraging. They would check in on me and celebrate small achievements, which made me feel valued.’ (P3, 33, female, urban)

‘A dedicated physiotherapist from Ikhwezi Lokusa Special School was my main support during the adjustment. She was incredibly patient and helped me a lot.’ (P2, 49, male, rural)

The future aspirations of prosthetic users in this study reflect a wide range of hopes, shaped by their individual experiences with prosthetics and personal circumstances. Some participants were driven by the desire to reclaim physical abilities and pursue athletic achievements. P1, for example, had a dream of running again, motivated by their pre-amputation jogging routine. This activity, which once brought them stress relief, now served as a symbol of progress and determination. Their focus was on strengthening their balance and endurance, with the long-term goal of acquiring a specialised prosthesis designed for running. Similarly, P2, an aspiring athlete, had set their sights on representing South Africa (SA) in international sports competitions. Their ambition was clear in their proactive efforts to secure sponsorships and specialised training, highlighting how the prosthesis is not only a tool for mobility but a vehicle for reaching new levels of physical achievement:

‘One of my big dreams is to start running again. Before my amputation, I used to jog to relieve stress, and I’d love to do that again. I’d be so happy to get a prosthesis that would allow me to run like Oscar Pistorius.’ (P1, 27, female, rural)

‘I’m hoping to get a prosthesis that is specifically designed for sports, so I can perform at my best. I often see other athletes with prosthetics built for both walking and sports, and that’s what I aspire to have.’ (P2, 49, male, rural)

For other participants, future aspirations were more closely tied to career opportunities and personal independence. P3 viewed the prosthesis as an enabler for engaging in meaningful work, particularly in healthcare or education, where they hope to use their experiences to inspire and support others. This reflects a broader sense of purpose, where prosthetics are seen as a tool for contributing to the community. Participant 4, on the other hand, aspired to return to their previous job, a source of stability and pride:

‘I see my prosthesis opening up job opportunities for me in the future.’ (P3, 33, female, urban)

‘I hope to return to my previous job with the help of the prosthesis.’ (P4, 45, male, rural)

## Discussion

The discussion is structured according to the three themes of this study, which showcase the experiences of lower limb prosthetic users in coping and adjusting to prosthesis use in the OR Tambo District.

### Facing psychological and identity adjustments

The participants revealed a multifaceted journey of facing emotional and physical challenges (including mobility limitations), which hinder psychological and social adjustments to limb loss. Their initial challenges included psychological responses or reactions following receiving a prosthesis. These responses to prosthesis use were marked by shock, sadness and an overwhelming feeling of loss, as many participants struggled to accept their new reality. Given that the participants worried about their mobility and functionality, these responses were also linked to what they perceived as having to be reliant and dependent on others for their basic needs following the limb loss or assistance from others to perform everyday activities – a factor that brings about feelings of disempowerment. This finding is consistent with prior research on the psychological impact of amputation (Dadkhah et al. 2013; Pedra et al. 2018). Like these findings, this impact reveals that the emotional challenges of losing a limb can include feelings of grief, loss and a disruption of self-identity (Murray & Forshaw 2013). Such findings foreground the need for consistent psychological support as an integral part of the prosthetic rehabilitation process and practice, especially in the initial stages of prosthetic rehabilitation. Providing access to counselling or peer-support services could greatly aid in helping users process these emotions of loss. Ostler et al. (2014) have argued that integrating psychological services into the standard care process would ensure that users have a safe space to address these feelings and develop a positive mindset, a factor shown to enhance adaptation outcomes.

Following a space where the users initially did not regard this assistive technology as a sufficient resource for improving their functional capacities, they also acknowledged and celebrated achievements within this journey. They referred to achievements as personal milestones and progress experienced by prosthesis users as they adjusted to life with a prosthesis. Worth noting, we see that these achievements are not simply outcomes but represent significant steps in coping and adapting, such as regaining mobility and achieving functional independence. In this case, the prosthesis serves as an enabler for empowerment in this post-amputation rehabilitation, with mobility being integral to regaining independence. These are key factors that are thought to enhance well-being in conceptual models of rehabilitation, such as the *International Classification of Functioning, Disability and Health* (World Health Organization [WHO] 2001).

Assistive technologies help people maintain or improve functionality (Pousada-Garcia et al. 2021), but the participants’ experiences show that the benefit extends beyond functional capabilities. This signifies a profound meaning of what prosthetics enable. The participants in this study reported that while they initially felt incomplete or diminished because of losing a limb, with time, they embodied the prosthesis into their new identity and regained confidence and independence. Additionally, participants expressed how the prosthesis improved their self-esteem and body image, findings that are mirrored in work by Kizilkurt et al. (2020). Kizilkurt et al.’s study found that improved mobility and independence through prosthesis use contributed to positive self-perception and body image recovery, particularly in the long term. These findings uncover novel contributions around prosthesis embodiment. While previous studies such as Murray and Forshaw (2013) have explored identity reconstruction following limb loss, this study offers a rural South Africa perspective, where cultural beliefs, social stigma and environmental barriers strongly influence how prosthetic users embody their devices and adjust to new identities.

The participants also expressed how they found a sense of relief and joy as they began to regain a sense of independence and mobility. It is this regained functional independence that facilitated emotional adjustment. Thus, it can be argued that, for these participants, physical functioning and independence were important benefits afforded by a prosthesis and influenced how they perceived their self-image (Sinha et al. 2014).

### Navigating daily societal realities and perceptions

A common finding relates to the alienation that some participants faced as a result of encountered stigma. Social interactions significantly influenced participants’ experiences of adjusting to prosthesis use, with some facing stigma. The stigma originated in negative societal attitudes, such as being stared at or ridiculed, and often led to social withdrawal for several participants. As Mireille and Foje (2019) posited, social stigma surrounding visible prosthetics can create feelings of isolation and exacerbate the emotional burden of prosthesis users. To address societal stigma, community education programmes on assistive technologies are crucial. This may include educating the public on amputation and prosthesis use to reduce discrimination and foster a more inclusive environment for prosthetic users. Community awareness campaigns could also be beneficial in reframing prosthetic use as a step towards independence and resilience, not a source of stigma.

Conversely, other participants in this study reported positive interactions, where community members admired their resilience and supported their efforts to reintegrate. This confirms findings by Resnik, Borgia and Silver (2017), which emphasise that community support and positive social interactions are crucial for prosthetic users’ psychological well-being and social reintegration. Such findings also foreground the importance of educating communities. Positive community engagement not only mitigates the impact of stigma but also fosters confidence and a sense of belonging, as seen in several participants’ experiences. Falgares et al. (2019) emphasised that family support helps users build self-confidence and maintain motivation during the rehabilitation process. This observation further highlights the critical role of family involvement in the rehabilitation process (Lee et al. 2023). The emotional encouragement and practical help provided by family members can significantly reduce the stress and fears associated with adjusting to a prosthesis and enhance overall quality of life, especially in the absence of the often-needed psychological support.

Beyond personal challenges, the participants also encountered environmental challenges related to navigating uneven rural terrain, which evoked feelings of frustration and setbacks in their prosthetic rehabilitation. These experiences confirm Razak et al.’s (2016) findings, which showed how environmental factors, such as poor infrastructure and rugged landscapes, can hinder prosthetic users’ mobility, particularly in rural areas. While it may be the case that the commonly accessible designs within public healthcare facilities are those that are not appropriate for rural settings, it is also the case that the rehabilitation process often does not take place in such simulated infrastructure but in well-built rehabilitation facilities, and this may not fully prepare users for what they will confront at home. There is therefore a need for prosthetic designs to effectively accommodate environmental challenges specific to rural settings, where infrastructure may not be conducive to mobility when using a prosthesis. Developing prosthetics specifically designed for rural environments (such as those with better stability and durability) could help alleviate some of these difficulties.

### Learning to cope and receive support

Using a prosthetic requires physical strength and resilience, as users must continuously adapt to the device. Physical discomfort, such as pressure sores and other complications, further impeded the participants’ adjustment process. According to Dakhil et al. (2019), physical issues such as prosthesis-related skin problems are common, particularly when prosthetic users have limited access to follow-up care. The participants in this study faced similar challenges, as access to consistent healthcare services in the rural OR Tambo District is limited, forcing many to self-manage their complications. This reflects broader healthcare system access issues in low-resourced settings, as Harkins et al. (2013) highlight. The importance of ongoing medical support for prosthetic users is integral to preventing and managing secondary complications. Perhaps implementing a mobile clinic or community-based follow-up programmes could help bridge this gap in rural areas (Harris et al. 2021). Implementing these would also help with ensuring that users receive timely adjustments and advice related to the issues they are faced with. Regular follow-ups would also allow prosthetists to monitor and address these issues proactively, ultimately improving comfort and reducing the risks.

Despite these physical and environmental challenges, a key finding is that participants developed strategies to cope, such as using walking aids or adjusting their routines. This is consistent with findings from Falgares et al. (2019), who noted that adaptive coping strategies are essential for long-term success in prosthetic rehabilitation. Participants who had better access to rehabilitation services reported better physical and emotional outcomes, reflecting the importance of professional support in prosthetic adjustment. The critical role of professional rehabilitation services is affirmed by Dadkhah et al. (2013) for enhancing both mobility and emotional adjustment in prosthetic users. However, for many participants, formal support services are limited, requiring them to rely heavily on self-directed learning and dependency on family for assistance. Prosthetic users in rural and underserved areas particularly often lack consistent access to rehabilitation services, increasing their reliance on self-management and informal support networks (Harkins et al. 2013). It is here that peer-support programmes in the community may be helpful in closing the gap. In the United States, individuals with lower limb loss reported positive experiences about their prosthetic legs and viewed peer support as a helpful source for both information and emotional support, potentially benefitting functional and psychological recovery after amputation (Lee et al. 2024). Similar benefits of peer support have been found in SA in mental health services (De Wet, Sunkel & Pretorius 2022). Likewise, family support emerged as a key factor in participants’ ability to cope, with participants frequently citing emotional and practical help from family members as crucial in their adjustment. This finding is consistent with Stuckey et al. (2020), who found that family involvement is associated with better emotional well-being and functional outcomes in prosthetic users. While there is still a long way towards achieving successful inclusion of formal peer support within healthcare services in the South African context, practitioners should continue advocating for these services as part of community-based rehabilitation services.

## Conclusion

This study highlights the complex and deeply personal journeys of lower limb prosthetic users in the OR Tambo District, emphasising the multifaceted challenges and achievements they experience in adjusting to prosthesis use. The experiences of lower limb prosthetic users in this study reflect the complex interplay of emotional, social, physical and environmental factors in the adjustment process. Emotional resilience, positive societal attitudes and strong support systems are critical to successful adaptation. However, the challenges of limited healthcare access, stigma and environmental barriers highlight the need for more targeted interventions, particularly in rural areas. These findings emphasise the importance of developing Medical Orthotists and Prosthetists (MOPs) services that extend beyond device provision to include continuous support and follow-ups. Future efforts should focus on improving access to rehabilitation services, reducing stigma through community education and designing prosthetic solutions that better address the unique needs of users in rural contexts.
